# In Vitro Evaluation of the UVCeed Mobile Disinfection Device: A Rapid, Portable Approach for Surface Sterilization

**DOI:** 10.7759/cureus.80601

**Published:** 2025-03-15

**Authors:** Mitchell K Ng, Michael A Mont, Peter M Bonutti

**Affiliations:** 1 Orthopaedic Surgery, Maimonides Medical Center, Brooklyn, USA; 2 Orthopaedic Surgery, LifeBridge Health, Baltimore, USA; 3 Orthopaedic Surgery, Sarah Bush Lincoln Bonutti Clinic, Effingham, USA

**Keywords:** artificial intelligence, augmented reality, disinfection efficiency, ultraviolet c, uvceed

## Abstract

Introduction*: *Ultraviolet-C (UVC) sterilization technologies have garnered significant attention for their potential to inactivate pathogens. The UVCeed device, a smartphone-enabled UVC sterilization tool, offers a portable and user-friendly option for disinfection. This study investigates the disinfection efficacy of UVCeed against bacterial and viral pathogens, including *Staphylococcus aureus*, *Escherichia coli*, *Klebsiella pneumoniae*, and* SARS-CoV-2*.

Methods*: *Pathogens were inoculated onto sterile surfaces such as urine cups (bacteria) or glass slides (virus) and exposed to the UVCeed device at controlled distances (65 mm, 100 mm, and 165 mm) and durations ranging from 0 to 64 seconds. Laboratory conditions were standardized at 72°F and 40-50% relative humidity. Assessment methods included viable plate counts for bacterial colonies and cytopathic effect (CPE) measurements for *SARS-CoV-2* using Vero E6 cell cultures. Crystal violet staining confirmed viral cell viability post-exposure. Key metrics, such as log reductions in pathogen concentration, were calculated to quantify disinfection efficacy.

Results*: *UVCeed achieved significant pathogen reductions, with log reductions exceeding 6.0 for *Staphylococcus aureus*, *Escherichia coli*, and *Klebsiella pneumoniae *after 64 seconds at 65 mm. For *SARS-CoV-2*, viral concentrations decreased by over four log-units within the same exposure time. Threshold times for effective disinfection varied by distance, with shorter distances achieving reductions faster. Compared to Lysol® wipes, which required up to four minutes of wetness for similar effectiveness, UVCeed disinfected a 6”x 6” surface in ~15 seconds and an 8”x 8” surface in ~37 seconds.

Discussion*:* These results highlight UVCeed’s efficiency as a rapid and portable disinfection solution. Its ability to inactivate pathogens within seconds makes it a valuable alternative to chemical disinfectants, especially for high-touch surfaces. The device’s efficacy varied with distance and exposure time, underscoring the importance of proper usage. Future research should explore real-world applications and expand pathogen testing to validate these findings further.

## Introduction

Surface disinfection is a critical component of infection prevention, particularly in high-touch areas prone to contamination by pathogens such as bacteria, viruses, and fungi [1]. These microorganisms can survive on surfaces for extended periods, acting as reservoirs for disease transmission through contact or cross-contamination [2]. The COVID-19 pandemic underscored the importance of robust disinfection practices in homes, workplaces, and public spaces, highlighting the need for innovative, efficient, and sustainable solutions [3,4].

Chemical disinfectants, including sprays, wipes, and alcohol-based sanitizers, are the most commonly used methods for surface cleaning [5,6]. While effective, these products have notable limitations. Improper application, such as insufficient contact time or uneven coverage, can render them ineffective, leaving pathogens on surfaces [7]. Harmful residues from chemical disinfectants pose additional risks, especially in environments with children, pets, or food preparation areas. Prolonged exposure to disinfectant chemicals has been linked to health issues, including skin irritation, respiratory problems, and potential neurological effects [8]. Furthermore, the environmental impact of single-use disinfectants, including non-biodegradable wipes and plastic packaging, contributes to growing concerns about sustainability and waste management [9].

To address these challenges, ultraviolet-C (UVC) technology has emerged as a chemical-free alternative for disinfection [10,11]. Operating within the 200-280 nm wavelength range, UVC light effectively neutralizes microorganisms by disrupting their DNA or RNA, preventing replication [12]. Unlike chemical disinfectants, UVC leaves no residues, is reusable, and is safe for use on various surfaces, including food-contact areas [13]. UVC technology offers a sustainable solution that minimizes health risks and environmental impact [14]. Despite these advantages, conventional UVC systems often face challenges, such as ensuring consistent surface coverage and achieving precise exposure times [15].

The UVCeed mobile UVC disinfection device addresses these limitations with a range of unique innovations [16]. By integrating augmented reality (AR), artificial intelligence (AI), and gamification, UVCeed enhances the disinfection process in several key ways. AR provides real-time visual guidance, allowing users to identify treated and untreated areas for thorough coverage [11]. AI optimizes UVC dosage based on surface type, distance, and user movement, ensuring effective pathogen inactivation. Gamification elements, such as visualized pathogen reduction, engage users and encourage adherence to proper disinfection practices. Safety is also prioritized, as the device features machine vision to detect humans or pets and automatically disable UVC output to prevent accidental exposure [11]. These features, combined with its portability and intuitive smartphone interface, position UVCeed as a transformative tool in surface disinfection.

Given these unique potential advantages, the aim of this paper was to evaluate the efficacy of UVCeed in reducing pathogen concentrations in vitro, including bacteria and viruses, under controlled conditions. We also explored its innovative features, benchmarked it against traditional disinfectants, and assessed potential real-world applications.

## Materials and methods

Experimental Setup

The UVCeed mobile UVC disinfection device (Bonutti Technologies, Effingham, IL) is a smartphone-mounted UVC exposure system that operates through a Bluetooth-linked application. The device was tested for its efficacy against bacterial pathogens (*Staphylococcus aureus, Escherichia coli, and Klebsiella pneumoniae*) and the viral pathogen *SARS-CoV-2*. Experiments were conducted at Spectral Platforms facilities under controlled laboratory conditions at 72°F and 40-50% relative humidity to ensure consistency.

Pathogen inoculum preparation

For bacterial tests, inocula were prepared by growing high concentrations of test organisms, approximately 10⁸-10⁹ colonu-forming unit (CFU)/mL) in saline solutions. The bacterial concentration in the inoculum was determined through serial dilution of the test inoculum, followed by plating 0.1 mL of each dilution onto blood agar plates. The plates were incubated for 24 hours to allow colony growth. Controlled amounts of the inoculum (0.1 mL) were then placed in sterile urine cups to serve as test samples. These test samples were exposed to the UVC air device under controlled conditions, including specific distances and exposure durations as detailed in the following section of the disinfection protocol. To assess bacterial concentration on the exposed samples, the samples were washed with 1X saline solution, and serial dilutions of the wash solution were prepared. A volume of 0.1 mL from the wash solution and its dilutions was plated on blood agar plates for further incubation. The bacterial concentration of the original test samples was calculated based on the number of visible colonies, adjusted using the appropriate dilution factors and test volumes.

Viral samples were prepared by incubating *SARS-CoV-2* in Vero E6 cell cultures to achieve a concentration of approximately 10⁶ plaque-forming unit (PFU)/mL. The prepared inocula were then distributed onto sterile urine cups (bacteria) or glass slides (virus) for testing. To assess the reduction in viral loads, all shell vials were incubated at 35°C for 72 hours and analyzed for cytopathic effects (CPE) using a bottom-illuminated microscope. Damaged cells appeared as dark spots, and CPE was quantified using a scoring system ranging from 0 (no CPE) to 4 (maximum CPE). Direct observation of CPE has limitations in reliability, as the dark spots from damaged cells can be missed due to depth-of-focus issues. Therefore, trends observed using this method should be corroborated with alternative approaches. The threshold time for *SARS-CoV-2* aligns closely with that observed for *E. coli and S. aureus*.

Disinfection Protocol

Test samples were placed at varying distances (65 mm, 100 mm, and 165 mm) from the UVCeed device and exposed for durations ranging from 0 to 64 seconds. Control samples were maintained under identical conditions without UVC exposure. Post-exposure, bacterial samples were washed, diluted, and plated on blood agar for colony enumeration. Viral samples were evaluated using cytopathic effect (CPE) assays, crystal violet staining, and impedance-based cell analysis to quantify viral load reductions.

Surface Wipes Experiment

All experiments were conducted in a controlled indoor environment with a stable room temperature of 72°F and relative humidity maintained between 40% and 50%. Air vents cycled 2 to 4 times during each measurement period to ensure consistent conditions. A dark, non-porous table surface was selected for testing, with two square areas (6”x 6” and 8”x 8”) demarcated using painter’s tape to ensure uniformity across trials. Lysol® disinfecting wipes (Reckitt Benckiser plc) were used for all tests, and surface drying times were assessed through visual observation under consistent lighting and tactile confirmation using the finger swipe method. For each area, a single application of the wipe was used to cover the entire surface, and a stopwatch was started immediately to record the time until 50% and complete dryness. These measurements provided a basis for comparing drying dynamics between the two areas.

To evaluate surface wetness maintenance, each test area was wiped thoroughly, and a stopwatch was used to monitor a 4-minute duration. Visual and tactile methods were employed to detect drying, and the surface was rewiped with the same Lysol® wipe whenever drying was observed. The number of reapplications required to maintain wetness over the 4-minute period was recorded for both areas. Additionally, disinfection times for the UVCeed device were calculated by interpolating log reductions of *E. coli and S. aureus *using a dose-response curve created through viable plate count methods. All results were documented and repeated under identical conditions to ensure consistency and reliability. This approach ensured a comprehensive evaluation of both drying times and the ability to maintain wetness for effective disinfection.

Data analysis

Log reductions in pathogen concentrations were calculated by comparing exposed samples to controls. Data trends, such as threshold exposure times and log-linear reduction rates, were analyzed to evaluate disinfection efficacy across varying distances and durations. The statistical significance of findings was confirmed through repeat trials and consistency checks.

The study adhered to strict validation criteria, ensuring that control samples consistently yielded positive results and that experimental samples demonstrated clear reductions in pathogen concentrations. Results from bacterial and viral tests were cross-validated through multiple quantitative and qualitative assessment methods to ensure reliability. Although no regulatory standards explicitly define passing criteria for UVC disinfection devices, reductions exceeding 4 log-units in viable pathogen concentrations were considered significant. The device's efficacy was assessed based on observed pathogen reductions, user safety features, and operational reliability.

## Results

The efficacy of the UVCeed sterilization device was evaluated against bacterial pathogens (*Staphylococcus aureus, Escherichia coli, and Klebsiella pneumoniae*) and viral pathogens (*SARS-CoV-2*) across multiple testing scenarios. Results consistently demonstrate the device's ability to significantly reduce pathogen concentrations under controlled conditions of distance and exposure time.

Reduction in bacterial pathogen concentrations

*Reduction in the Concentrations of* *Staphylococcus aureus *

Log reductions were observed after a threshold exposure time, with linear increases in pathogen reduction over log-transformed exposure times. At a distance of 65 mm, the device achieved complete inactivation (>6-log reduction) within 64 seconds of exposure. Similar trends were observed at exposure distances of 100 mm and 165 mm, albeit requiring longer exposure times.

*Reduction in the Concentrations of* *Escherichia coli*

A similar reduction trend was observed, with significant log reductions at all tested distances (65 mm, 100 mm, and 165 mm). Maximum reduction (>6-log) was achieved within 64 seconds of exposure at a distance of 65 mm.

*Reduction in the Concentrations of​​​​​​​* *Klebsiella pneumoniae*

**Figure 1:**
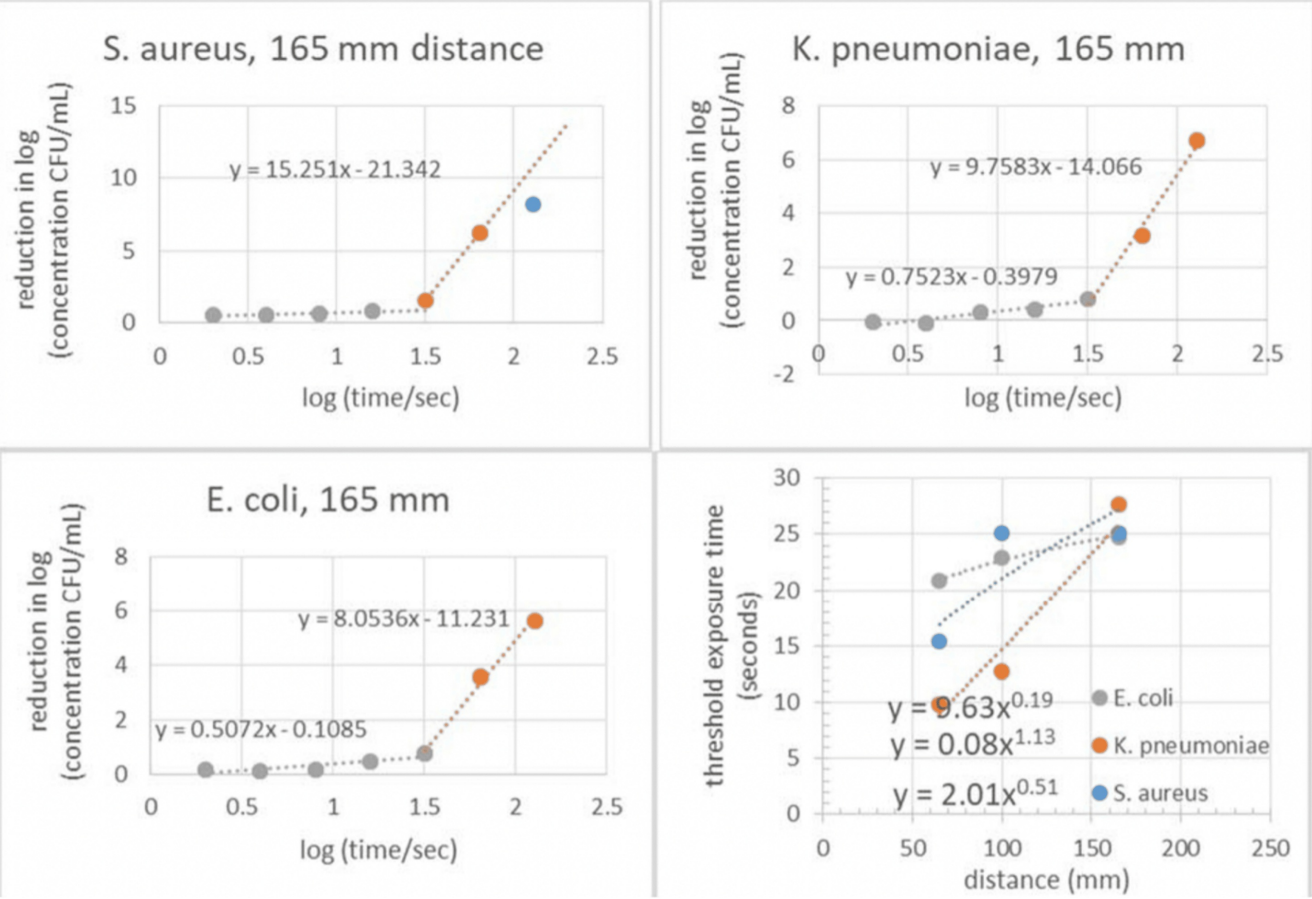
Clockwise from bottom left. Reduction in pathogen concentration for *E. coli​​​​​​,* *S. aureus, K. pneumoniae* and variation of exposure threshold with exposure distance.

Results mirrored those of *E. coli and S. aureus*, with progressive log reductions following threshold exposure times. Maximum pathogen reductions were recorded at closer distances and longer exposure durations (Figure 1).

Reduction in viral pathogen concentrations

Reduction in the Concentrations of​​​​​​​ SARS-CoV-2

**Figure 2:**
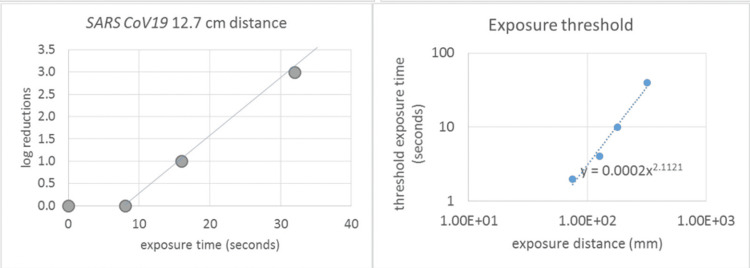
Reduction in pathogen concentration Reduction in pathogen concentration for *SARS CoV-2*, detailing the variation of exposure threshold time with exposure distance.

The device achieved significant reductions in viral loads (>4-log reduction) after 64 seconds of exposure at a distance of 65 mm (Figure 2).

A threshold exposure time (~16 seconds) was required to initiate measurable reductions in viral concentrations. Beyond this threshold, pathogen reduction increased linearly with exposure duration. 

Threshold and scaling behaviors

A threshold exposure time was consistently observed for all tested pathogens, below which pathogen reductions were minimal. Once this threshold was exceeded, reductions followed a predictable linear relationship with log-transformed exposure times. The effectiveness of the device was inversely proportional to the distance between the UVC source and the test sample, with closer distances yielding faster and greater pathogen reductions.

Benchmarking against traditional disinfectants, comparative testing against Lysol® wipes demonstrated UVCeed's superior speed and efficacy (Table 1). Lysol® wipes required sustained wetness for four minutes to achieve 99.9% disinfection, while UVCeed achieved a 99.99% pathogen reduction in just 15.28 seconds for a 6”x 6” surface and 36.61 seconds for an 8”x 8” surface. Wipes dry within 52-65 seconds, necessitating up to seven reapplications to maintain surface wetness over four minutes. In contrast, UVCeed provided rapid and residue-free disinfection without requiring repeated applications, making it safer and more practical for frequent use, especially around food-contact areas.

**Table 1:** Summary of drying time measurements and reapplication counts.

Test area	Time to 50% dryness	Time to complete drying	Number of reapplications to maintain wetness for 4 min, required for 99.9% disinfection	Time for 99.99% disinfection using UVCeed
6” x 6”	52 seconds	1 minute 58 seconds	7	15.28 seconds
8” x 8”	1 minute 5 seconds	2 minutes 28 seconds	5	36.61 seconds

## Discussion

The aim of this study was to evaluate the efficacy of UVCeed device to reduce bacterial and viral pathogen concentrations on surfaces. Our results demonstrate UVCeed was able to achieved >6-log reductions in common bacterial pathogens, including *Staphylococcus aureus, Escherichia coli, and Klebsiella pneumoniae*, within 64 seconds of exposure at 65 mm. In addition, UVCeed successfully inactivated* SARS-CoV-2*, achieving >4-log reductions in viral loads [17]. Overall, our results highlight the potential utility of UVCeed as a disinfection method, particularly in environments that require frequent and thorough cleaning.

Relative to conventional chemical disinfectants (e.g., Lysol® wipes), UVCeed offers several distinct advantages. First, while disinfectant wipes may require prolonged wetness on an appropriate surface of up to four minutes to achieve 99.9% disinfection, our data demonstrates that UVCeed achieved 99.99% disinfection within 15-37 seconds. This rapid time to disinfection, combined with its lack of residue after application [14,15], highlights UVCeed as a promising alternative, particularly in settings where potential chemical residues pose health or safety concerns (e.g., food-contact areas, homes with children or pets, and healthcare facilities) [5]. In addition, unique to UVCeed relative to other UVC technologies is its portability and smartphone integration, which further enhance ease of use across a wide variety of environments [18].

The study also highlights the importance of operational parameters, such as exposure time and distance, in determining the efficacy of UVCeed. Compared to traditional chemical disinfectants, such as Lysol® wipes, which require up to four minutes of wetness for comparable efficacy, UVC technology provided significant pathogen reductions in a fraction of the time. Overall, closer distances yielded faster and more substantial pathogen reductions, emphasizing the need for proper usage guidelines to optimize performance. These findings are consistent with the established principles of UVC disinfection, where the intensity of UVC radiation diminishes with increasing distance [19,20]. The threshold times observed in our study further highlight the importance of maintaining appropriate exposure durations to achieve effective disinfection. 

One of the key characteristics of UVCeed is its incorporation of augmented reality (AR), artificial intelligence (AI), and gamification [17]. These features not only ensure thorough and accurate disinfection in a real-world setting but also enhance user engagement and compliance. The AR visual guidance for identifying treated and untreated areas [21], combined with AI-optimized dosage adjustments, addresses common limitations associated with traditional UVC systems, including uneven surface coverage and inconsistent exposure times [22]. In addition, the safety mechanisms of the UVCeed, including automatic shutdown upon detecting human presence, adds an additional layer of user protection across a variety of real-world environments, both in and outside of hospitals [23]. 

Despite these promising findings, the study has several limitations. All experiments and pathogen testing were conducted under controlled laboratory conditions, which may not fully replicate real-world environments where variables such as surface texture, ambient lighting, and airflow can potentially impact efficacy. In addition, our study only included a limited number of bacterial and viral pathogens; expanding the scope to include other common microorganisms would more fully characterize the potential of UVCeed. Finally, the long-term durability and consistent performance of the UVCeed device were not evaluated, which are potentially important factors for practical and frequent use in a real-world setting. Addressing these limitations in future studies will strengthen the generalizability and applicability of the findings. Nevertheless, this study serves as the first, to the best of our knowledge, to characterize the effectiveness of a novel UVC sterilization technology against bacterial/viral pathogens.

## Conclusions

This study demonstrated the effectiveness of UVC sterilization technology as a rapid and efficient tool for disinfection against bacterial pathogens (*Staphylococcus aureus, Escherichia coli, Klebsiella pneumoniae*) and the viral pathogen *SARS-CoV-2*. Under controlled laboratory conditions, UVC exposure achieved over 6-log reductions for bacterial pathogens and more than 4-log reductions for *SARS-CoV-2* within 64 seconds at a distance of 65 mm. Across all aims, UVCeed consistently demonstrated rapid and effective pathogen reductions, achieving greater than 99.99% disinfection within seconds. Its innovative design, superior speed, and safety features make it a transformative solution for surface disinfection, outperforming traditional chemical disinfectants and other UVC technologies.

The integration of advanced features such as AR, AI, and gamification further enhances its utility and user experience. While the controlled laboratory results presented in this study are promising, further research is needed to validate the device’s performance in real-world settings and across a broader range of pathogens. With its innovative design and demonstrated efficacy, UVCeed has the potential to transform surface disinfection practices, providing a safer, more sustainable, and efficient solution for infection prevention across diverse environments.
