# Correction: In Vitro Evaluation of the UVCeed Mobile Disinfection Device: A Rapid, Portable Approach for Surface Sterilization

**DOI:** 10.7759/cureus.c251

**Published:** 2025-08-20

**Authors:** Mitchell K Ng, Michael A Mont, Peter M Bonutti

**Affiliations:** 1 Orthopaedic Surgery, Maimonides Medical Center, Brooklyn, USA; 2 Orthopaedic Surgery, LifeBridge Health, Baltimore, USA; 3 Orthopaedic Surgery, Sarah Bush Lincoln Bonutti Clinic, Effingham, USA

The title of this article has been corrected to read ‘In Vitro Evaluation of the UVCeed Mobile Disinfection Device: A Rapid, Portable Approach for Surface Sterilization’. The original version incorrectly included ‘Ultraviolet Ceed’ instead of ‘UVCeed’.

